# Transmission characteristics of tuberculosis in the high-TB-burden region of Southwest China

**DOI:** 10.3389/fpubh.2025.1670514

**Published:** 2025-11-19

**Authors:** Xing Yang, Haohao Ru, Tao Chen, Lianyong Chen, Qinxuan Ni, Shuangqun Yan, Rui Yang, Jinou Chen, Lin Xu

**Affiliations:** Yunnan Center for Disease Control and Prevention (Yunnan Academy of Preventive Medicine), Kunming, China

**Keywords:** *Mycobacterium tuberculosis*, whole genome sequencing, high burden setting, cluster analysis, transmission pattern

## Abstract

**Background:**

Whole genome sequencing (WGS) can provide valuable insights into the transmission patterns of *Mycobacterium tuberculosis*, which can inform effective intervention strategies for controlling tuberculosis (TB) in local areas.

**Methods:**

We conducted a retrospective study using strains isolated from L County between January 2019 and July 2020. Demographic data of patients were retrieved from National Tuberculosis Information Management System. A whole genome sequencing-based clustering analysis was performed to assess the transmission characteristics of pulmonary TB in L County. Univariate and multivariate logistic regression analyses were employed to identify potential risk factors associated with clustering.

**Results:**

A total of 143 strains from 136 confirmed TB cases were available for analysis, among them 74.26% of *M. tuberculosis* strains belonged to Lineage 2, while Lineage 4 comprised the reminder. Among the pulmonary TB cases, 38.97% (53/136) exhibited genomic-clustered strains (≤12 SNP threshold). Notably, 29 (62.26%) strains belonging to 15 clusters were isolated from the same township, while 10 (18.87%) strains belonging to 5 clusters originated from the same villages. The younger age group (≤44) exhibited a higher risk of clustering (*aOR* 19.21, 95% *CI*: 2.37–155.80, *p* = 0.006). Additionally, 9 (27.27%) drug resistant (DR) strains were identified within cluster, and 4 serial DR strains of 2 patients showed the accumulation of acquired drug resistance.

**Conclusion:**

A significant proportion of tuberculosis cases in L County are attributed to recent transmission occurring within communities or neighborhoods across townships for both sensitive and drug-resistant TB. These findings suggest that an active case-finding strategy should be implemented in this area to identify patients at the early stages. Furthermore, active screening should not only target key populations such as close household contacts or old population but also extend to all residents over 15 years. Improving chemotherapy treatment quality and patient follow-up is essential in L County.

## Background

Tuberculosis, caused by *Mycobacterium tuberculosis*, continues to pose a significant global public health threat. According to the Global Tuberculosis Report 2024, an estimate of 10.8 million new TB cases were reported worldwide, resulting in an incidence rate of 134 per 100,000 population. This reflects a sustained increase since 2021 and suggests that TB once again emerge as the leading cause of death from a single infectious agent (1). China ranked third among the 30 high-TB burdens countries, which highlighting ongoing challenges in the prevention and control of this disease (1).

The previous studies have demonstrated significant regional variations in the incidence and mortality of pulmonary tuberculosis (PTB) in China (2). Yunnan Province, located in the southwest of China, also bears a high burden of TB, the geospatial difference of TB prevalence distribution has been observed in Yunnan (3), and the current decline rate in this area is insufficient to meet the targets which is set by the World Health Organization (WHO) End TB Strategy (4, 5).

Controlling human-to-human transmission of TB is crucial for achieving the targets set by the WHO (6, 7). Prevention and control strategies that aligned with transmission dynamics could significantly accelerate the decline of this epidemic. However, due to the nature of the TB, traditional genotyping methods, including IS6110-RFLP, mycobacterial interspersed repetitive unit-variable number tandem repeats (MIRU-VNTRs) typing and spoligotyping, are insufficient for accurately decoding transmission patterns within populations, particularly in Beijing genotype strains are predominant areas, due to their low discriminatory power (8). Our previous molecular epidemiological study has established the predominance of the Beijing genotype across Yunnan province, however, it offered limited insights into the actual transmission networks (9).

WGS data can provide valuable insights into transmission dynamic, in which strains with near-identical genetic variants are considered to possess a transmission relationship (10, 11). However, previous WGS-based studies on TB transmission in China have predominantly focused on large cities characterized by high population density and frequent movement, such as Shenzhen (12). In contrast, rural areas of Yunnan, which are marked by geographical isolation and stable demographics, may exhibit distinct transmission patterns. Our study aimed to elucidate the dynamics of tuberculosis transmission in Yunnan’s region with the high TB burden through WGS analysis and generate evidence-based data to inform targeted TB control strategies in this endemic area.

## Methods

### Study population and setting

L County, located in northwestern Yunnan Province, served as one of 33 provincial-level drug-resistance surveillance sites in Yunnan. This mountainous region encompasses eight townships with 82.1% of residents live in rural mountainous areas (95% of the county’s terrain consists of high mountains and valleys). Furthermore, this county was the high incidence area of tuberculosis in Yunnan Province. We conducted a retrospective study using *Mycobacterium tuberculosis* strains collected in L County between January 2019 and July 2020. All confirmed TB cases of were consecutively enrolled for sputum culture at the laboratory of county-level hospital, culture-positive strains were subsequently transferred to the Tuberculosis Reference Laboratory of Yunnan province for further analysis. The demographic data of patients were retrieved from National Tuberculosis Information Management System. This retrospective study cause no harm to human subjects and involves no sensitive personal information or commercial interests, could be exempted from ethical review in accordance with the national legislation.

### Whole-genome sequence and bioinformatic analysis

Genomic DNA were extracted using the cetyl-trimethylammonium bromide-sodium chloride (CTAB) method (13). WGS was performed on the HiSeq (Illumina^1^) platform with an expected coverage of 100×, raw sequence data quality was assessed using FastQC (v0.12.0) (14), followed by adapter trimming and quality filtering, high-quality reads were aligned to the *M. tuberculosis* H37Rv reference genome (GenBank accession NC_000962.3) using BWA-MEM (v0.7.17). The low-quality SNPs and indels were filtered using bcftools (v1.21) with thresholds for minimum quality (Q ≥ 30), read depth (DP ≥ 5), and genotype call rate (≥90%). Variants with an alternate allele frequency (AF) < 0.75 were also excluded. Mutations included synonymous and nonsynonymous mutation, missense, frameshift mutations were annotated with SnpEff (v4.3t). Drug resistance and lineage were identified by TB-profiler (15, 16). Mutations matrix based on the mutation sites of each strain was generated by Snippy (v4.4.3), pairwise SNP distances were calculated using snp-dists (v0.7.0).

### Phylogenetic analysis

Based on the identified SNPs, a phylogeny tree was constructed with IQtree, using the maximum likelihood method with 100 bootstraps and visualized with iTOL (17). The minimum spanning tree was generated with Phyloviz. To minimize the potential distort of phylogenetic signals and transmission clustering, variations in known drug-resistant genes or intergenic regions, direct repeat regions, microsatellite-like sequences, transposition insertion sequences (such as IS6110), ESX secretion system protein genes, and PE/PPE/PGRS family genes were masked (18, 19). Transmission patterns were classified according to the phylogenetic clustering and geographic distribution. Strains sharing ≤12 SNPs between genomes (20–22) and originating from the same township were considered indicative of community transmission. Clustered strains from different townships were classified as cross-township transmission events.

### Data analysis

The clustering rate was calculated as following: (Number of clustered strains/Total number of sequenced strains) × 100%. We distinguished between drug-resistant tuberculosis (DR-TB) strains with acquired resistance and potential primary sensitive TB or DR-TB strains based on the presence of additional resistance-associated mutations. If strains within a cluster shared the same resistant mutation, we inferred that the emergence of drug resistant tuberculosis was attributable to the transmission of drug-resistant strains. Conversely, if the strains within a cluster exhibited different genotypic resistance and were isolated from previously treated patients, we considered it acquired drug resistance during transmission.

The distribution of continuous and categorical variables between groups was compared using the Wilcoxon rank sum test or the chi-square test when applicable. Logistic regression analysis was used to calculate the odds ratios (*OR*) and 95% confidence intervals (*CI*) for risk factors associated with genomic clustering. Variables with *p*-values less than 0.2 in the univariable analysis were included in the multivariable analysis to calculate the adjusted odds ratios (*aOR*). Factors with a *p*-value less than 0.05 in the final model were considered statistical significantly. All analysis were performed use SPSS (version17.0).

## Results

### Demographic characteristics of the patients

From January 2019 to July 2020, we collected 145 *Mycobacterium tuberculosis* isolates from 138 patients (6 patients provided multiple isolates), two isolates were excluded from subsequent analyses due to sequencing failure (*n* = 1) and contamination (*n* = 1). Finally, 136 patients (36.03% were smear-negative cases) included analysis. Among them, 75.0% were male, 96.32%were farmers, 67.65% were new cases, and the mean age ± standard deviation (SD) was 46.48 ± 14.30 years (Table 1).

**Table 1:** Demographic characteristics of the patients.

Demographic characteristic	New cases (%)	Retreated cases (%)	Total (%)
Gender (*N* = 136)			
Male	62 (45.59)	40 (29.41)	102 (75)
Female	30 (22.06)	4 (2.94)	34 (25)
Age group (*N* = 136)			
<18	3 (2.21)	0 (0)	3 (2.21)
18–44	40 (29.41)	15 (11.03)	55 (40.44)
45–64	35 (25.74)	24 (17.65)	59 (43.38)
≥65	14 (10.29)	5 (3.68)	19 (13.97)
Occupations (*N* = 136)			
Farmer	87 (63.97)	44 (32.35)	131 (96.32)
Others	5 (3.68)	0 (0)	5 (3.68)

### Drug resistant analysis

The genomic drug resistance prediction based on whole genome sequencing revealed that the overall drug resistance rate of 136 strains against 21 anti-tuberculosis drug was 24.26%. The rates of Rifampicin resistance (RR), multi-drug resistance (MDR), and extensive drug resistance (XDR) were 13.24, 4.41, and 1.47%, respectively. No resistance related mutations were found for new antituberculosis drugs.

### Phylogenetic analysis

**Figure 1:**
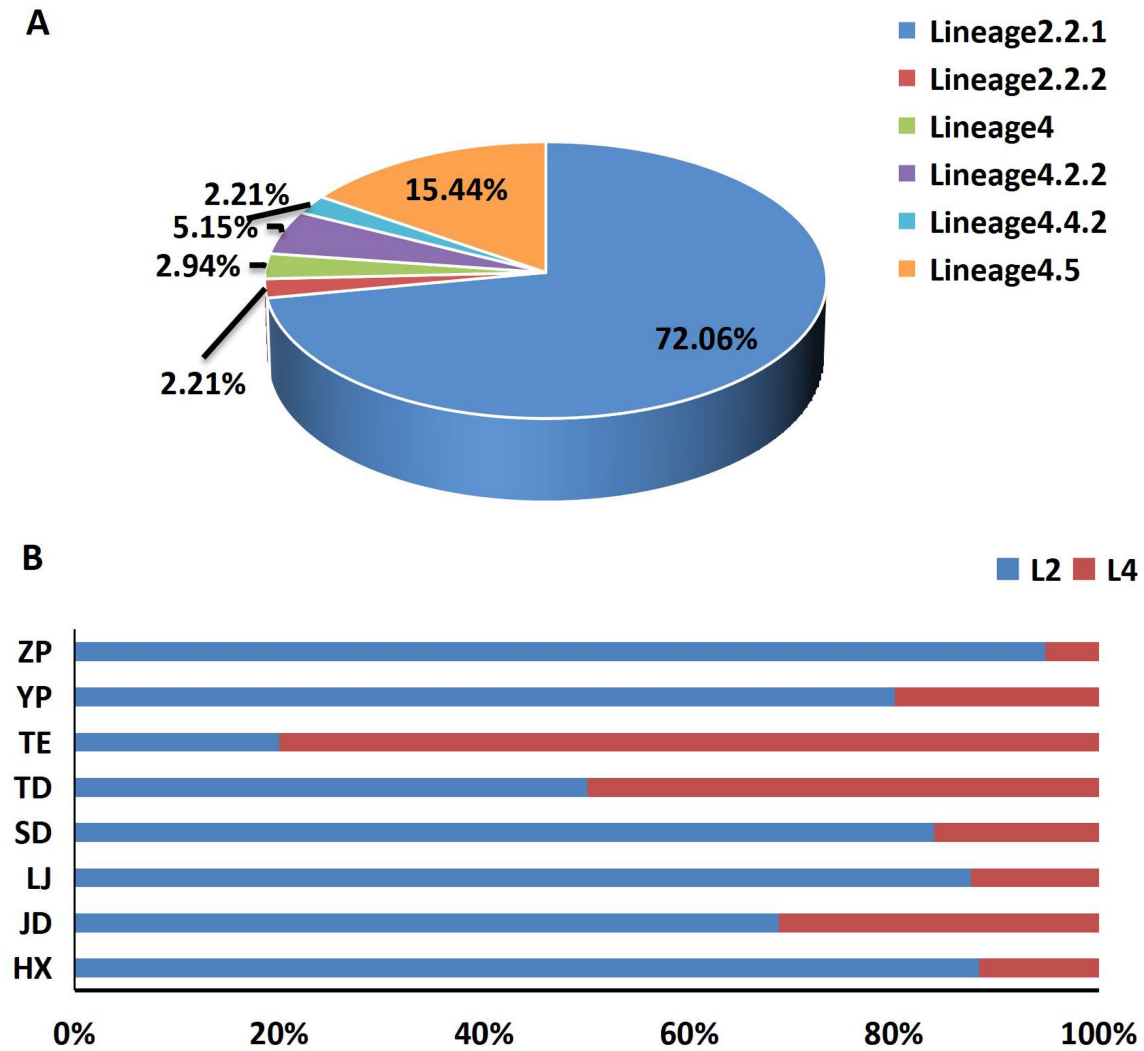
The composition and distribution of lineages. **(A)** The composition of different lineages and sub-lineages, **(B)** the main lineages distribution among 8 townships.

Among the total 136 strains, the majority of *M. tuberculosis* strains were belonged to the East-Asian lineage (Lineage2) (74.26%, 101/136), followed by the Euro-American lineage (Lineage4) (25.74%, 35/136), Lineage 2.2.1 (72.06%, 98/136) was the main sub-lineage among 136 strains, following by Lineage4.5 (15.44%, 21/136) (Figure 1A). A geographical divergence in lineage distribution was observed in different township, with Lineage 2 (Beijing genotype) ranging from 20 to 94.74%, and ZP township exhibiting the highest prevalence of this lineage. In contrast, Lineage 4 prevalence varied from 5.26 to 80%, with TE township showing the highest distribution (Figure 1B).

### The transmission clusters

**Figure 2:**
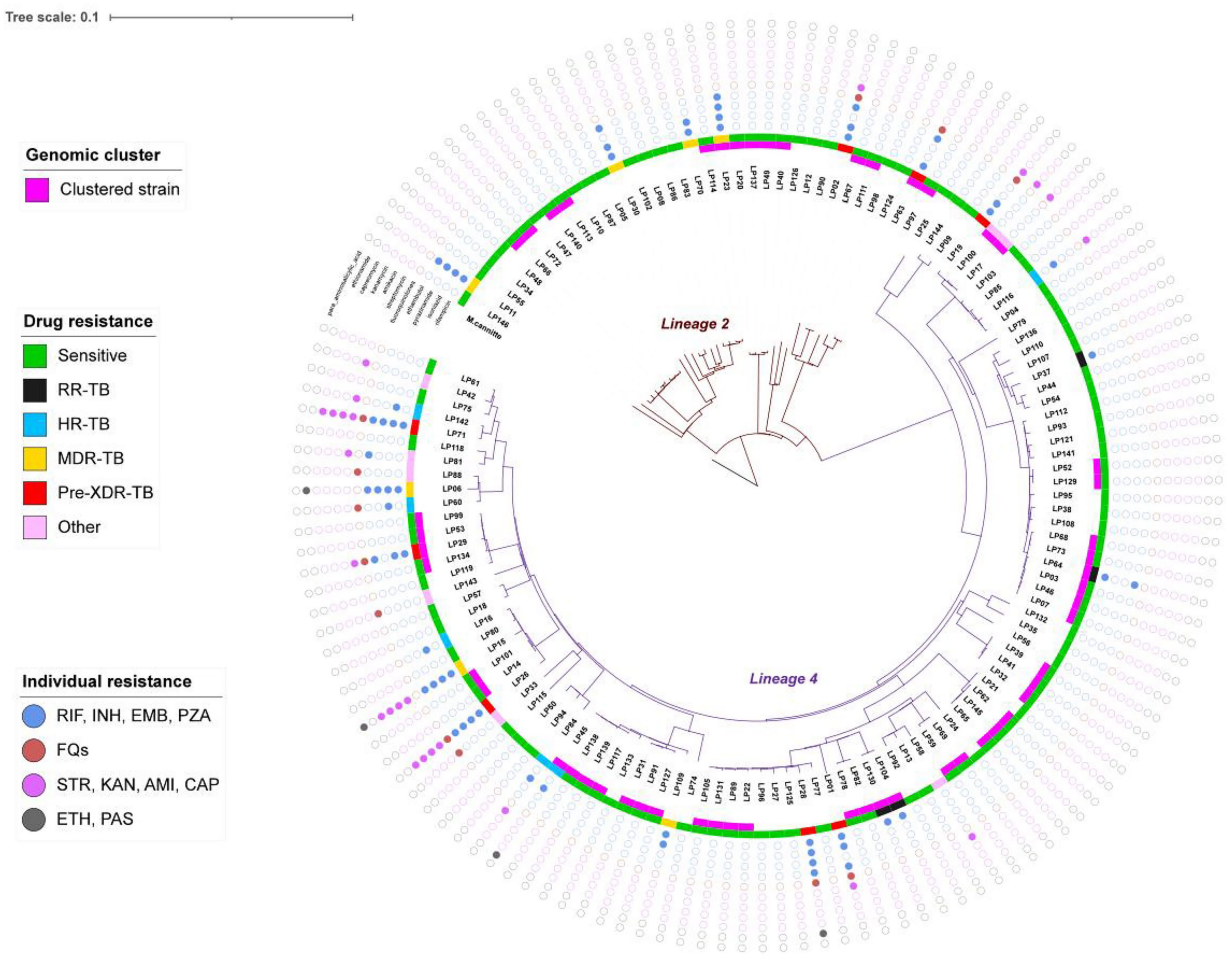
The maximum likelihood phylogeny tree of 136 strains. The inner branch indicates the genetic distance, the pink belt of inner circle indicates genomic-clustered strains differing by ≤12 single-nucleotide polymorphisms, the different color of middle circle indicates the drug resistant type and the outermost colored dots indicates the resistance to 11 anti-TB drugs. *Mycobacterium canettii* was used as an outgroup.

The maximum-likelihood phylogeny tree (Figure 2) showed limited diversity among L County. Fifty-three isolates grouped into 21 clusters, with a cluster rate of 38.97%, each cluster contained 2 to 6 strains. Among the clustered strains, 29 (54.72%) belonged to 15 clusters isolated from the same township, while 10 (18.87%) belonged to 5 clusters and were isolated from the same villages. The results of genomic clustering analysis suggested that recent transmission may have occurred among approximately 38.97% of patients in L County, with a majority of these transmissions were likely taken place within community or neighborhood.

**Figure 3:**
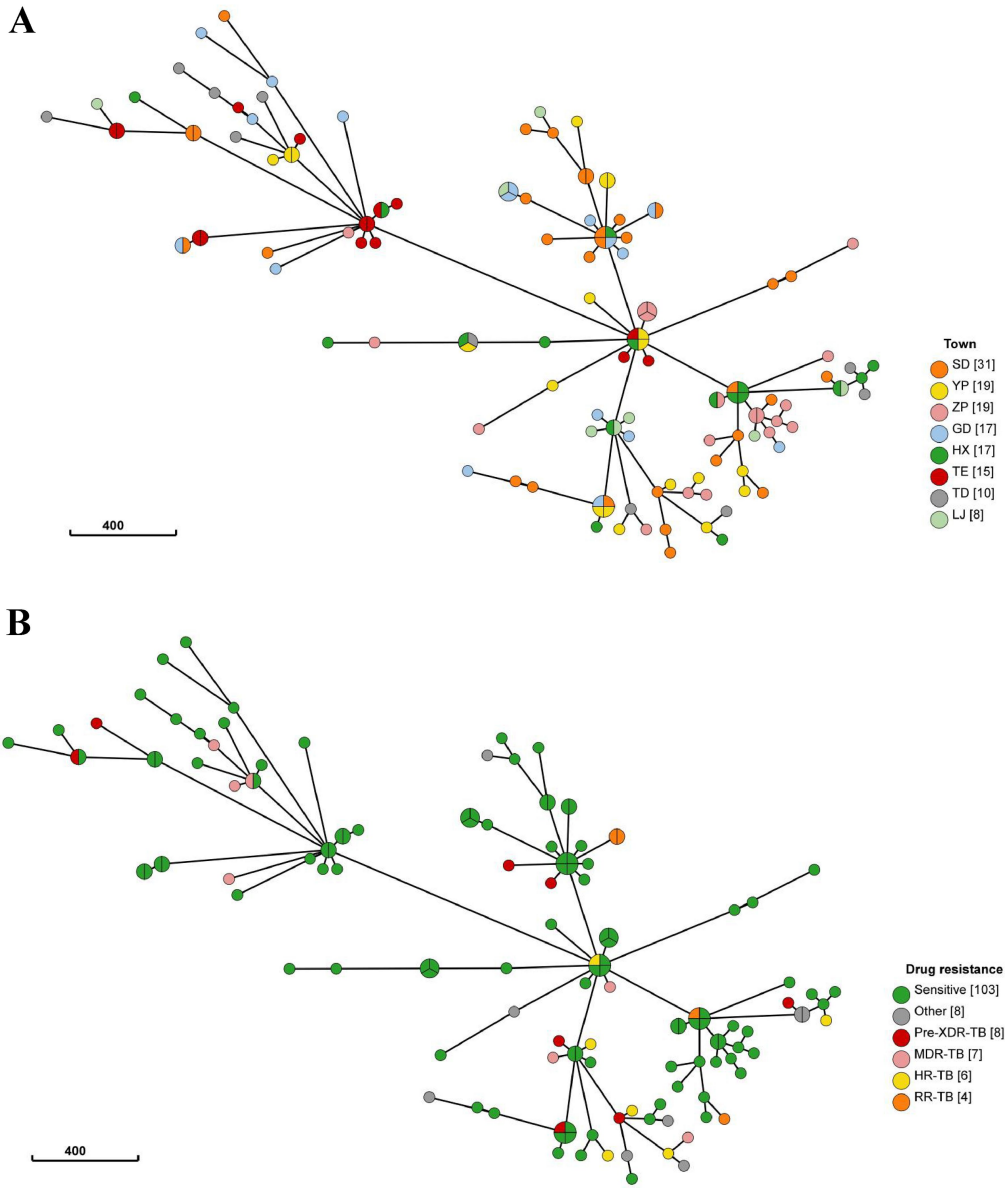
**(A)** The minimum spanning tree of 136 strains. The size of the circle represents the number of isolates in the cluster, the color of each circle represents the originated township of strain (TE, ZP, SD, YP were defined as high-burden townships), circle compromised of different color represents the cross-township transmission of this cluster. **(B)** The minimum spanning tree of 136 strains, The size of the circle represents the number of isolates in the cluster, the color of each circle represents drug resistance type of strains, circle compromised of non-green color represents the cluster contained drug-resistant strains.

To further investigate the characteristics of TB transmission in L County, we conducted a comprehensive analysis of the origins and drug resistance types of the clustered strains. Twelve clusters comprising 34 isolates were classified as cross-township transmissions, while 7 clusters with 15 isolates originated from distinct townships. Additionally, another 5 clusters consisting of 19 isolates included both intra- and inter-township cases, suggesting localized transmission with occasional spillover (Figure 3A). Furthermore, among the identified clusters, 7 contained drug-resistant strains (*n* = 9), the drug-resistant strains clustering rate was 27.27% (9/33). Notably, five clusters comprised both drug-resistant (*n* = 5) and drug-susceptible strains, those 5 drug-resistant strains within a cluster exhibited different genotypic resistance and were isolated from previously treated patients, we considered it indicative of resistance during transmission. Two clusters consisted solely of drug-resistant strains (2 strains per cluster), each strain of the same cluster shared with the incidental drug related mutations (2 strains with mutation of *rpoB* Ser450Leu, the other 2 with mutation of *rpsL* Lys88Met) profile indicating the primary transmission of resistance (Figure 3B), based on these, we can speculate that recent TB transmission in this area is active for both sensitive and drug-resistant forms.

### Longitudinal analysis of drug resistance acquisition

We obtained the drug resistance acquisition under the selection pressure of anti-tuberculosis drugs by analyzing 13 serial *Mycobacterium tuberculosis* isolates collected from six patients at various time points during anti-TB therapy. Three isolates (May 2019, May 2020, June 2020) from a previously treated but drug-sensitive patient exhibited no resistance mutations by WGS analysis. Six isolates from three initially drug-sensitive patients (2 new cases, 1 previously treated case) maintained susceptibility throughout standard therapy. In contrast, a retreated MDR-TB patient developed additional resistance to ethambutol (*embB* Met306Val) and pyrazinamide (*pncA* Gly24Asp) after 8 months of treatment with 2HRZE/10HRE. A new TB case with initial isoniazid (*katG* Ser315Thr) and streptomycin (*rpsL* Lys43Arg) resistance acquired aminoglycoside resistance (*rrs*_r.1401a > g) after 6 months treatment. This observation suggests that acquisition of drug resistance occurs may be more readily in DR-TB, particularly among those who received sub-optimal anti-tuberculosis therapy, the speculation based on this observation requires further verification.

### Factors associated with genomics clustering

In our analysis, we employed logistic regression to identify risk factors associated with clustering. The univariate analysis revealed that the less than 45 age group was significantly linked to clustering, and this association remained significant in the multivariate analysis. Compared to individuals over 65 years old, those in the younger age group exhibited a higher risk of clustering (*aOR* 19.21, 95% *CI*: 2.37–155.80, *p* = 0.006) (Table 2).

**Table 2:** Univariable and multivariable logistic regression of risk factors for genomic clustering.

Characteristic	Cluster rate (%)	Univariable regression		Multivariable regression	
		OR (95% CI)	*P*	aOR (95% CI)	*P*
Gender					
Male	42 (41.18)	1.46 (0.65–3.32)	0.362	..	..
Female	11 (32.35)	ref		..	
Age group					
≤44	30 (51.72)	19.29 (2.41–154.27)	0.005	19.21 (2.37–155.80)	0.006
45–64	22 (37.29)	1.80 (0.86–3.77)	0.118	1.61 (0.75–3.46)	0.223
≥65	1 (5.26)	ref		..	
Occupation					
Others	4 (80.00)	6.69 (0.73–61.6)	0.093	4.16 (0.42–40.80)	0.221
Farmers	49 (37.40)	ref		..	
Location					
High burden towns	34 (40.00)	1.12 (0.55–2.29)	0.751	..	..
Others	19 (37.25)	ref		..	
Previous TB					
Yes	15 (34.09)	0.74 (0.35–1.55)	0.42	..	..
No	38 (41.30)	ref		..	
Smear microscopy					
Positive	34 (39.08)	1.01 (0.49–2.08)	0.972	..	..
Negative	19 (38.78)	ref		..	
Drug resistant					
Yes	9 (27.27)	0.50 (0.21–1.19)	0.117	0.43 (0.18–1.07)	0.069
No	44 (42.72)	ref		..	
Main lineage					
L2	39 (38.61)	0.94 (0.43–2.07)	0.885	..	..
L4	14 (40.00)	ref		..	

## Discussion

Tuberculosis remains a significant public health challenge in China, exhibiting considerable variations in transmission characteristics across different regions. Our study presents the demographic characteristics, transmission dynamics, and acquisition drug resistance of *Mycobacterium tuberculosis* in Yunnan, which burdened by high rates of TB. Our findings provide an initial overview of the transmission patterns within this high-burden context, indicating a limited genetic diversity characterized exclusively by lineage 2 and lineage 4 strains. Our data specifically indicate that 74.26% of the strains belong to lineage 2, while 25.74% are classified as lineage 4. A significant proportion of the clustered strains (73.58%) is attributed to sub-lineage 2.2.1, which represents a modern sub-lineage within the Beijing family. These findings are consistent with previous reports that emphasize the predominance and widespread distribution of these lineages across China (23–25).

In L County, it was observed that 38.97% (53/136) of pulmonary tuberculosis cases exhibited genomic clustering within a period of less than 2 years. This clustering rate significantly exceeds those reported in Luo dian (13.1%) and Kashgar Prefecture (25.6%) from a 1-year study (24, 26), as well as surpassing the national average clustering rate of 23.0% (27). These findings indicate a significantly higher level of community transmission in L County compared to other regions of China. Several factors may contribute to this, first, L County experiences low population mobility while simultaneously experiencing a high prevalence of tuberculosis, coupled with the frequent gatherings that occur during traditional festivals, these factors create optimal conditions for airborne transmission of TB. Second, insufficient health awareness and limited access to healthcare services have resulted in prolonged diagnostic delays (exceeding 2 months) following the onset of symptoms, consequently, tuberculosis transmission due to these delays is significantly more pronounced in this region compared to other areas in Yunnan. Third, we observed suboptimal treatment outcomes in six cases examined, these instances of non-converted may contribute to ongoing transmission even after prolonged treatment duration.

According to the analysis of risk factors, younger individuals demonstrate a higher clustering rate compared to older adults (≥65), likely attributable to their increased social and occupational activities, which is consistent with findings from Shanghai (28). The disparities in lineage distribution highlight the necessity for region-specific strategies in tuberculosis control programs. Research conducted in Central Asia has revealed a significant association between Beijing strains and the spread of multidrug-resistant TB (MDR-TB) (29). Our study found Beijing genotype strains and the drug resistant were not independent risk factor for recent transmission, which contradicts findings from other studies conducted in China (26, 30). Compared to these mentioned studies, our sample size may have limited the statistical power necessary to detect transmission associated with Beijing strains, additionally, the inclusion of only a few clustered drug-resistant strains included could introduce bias when analyzing risk factors. Therefore, larger cohort studies are required to validate our current observations.

This study presents several limitations, primarily its retrospective design as opposed to a prospective approach, the absence of socioeconomic data for comprehensive analysis, and the lack of detailed epidemiological linkage data. Consequently, potential transmission clusters were inferred solely based on genetic distances among strains. Furthermore, accurately estimating the precise timing of potential transmission events presents significant challenges, clusters that originating years ago may still be propagating. The second limitation pertains sample size and composition, which may influence the interpret ability of our findings. During the study period, approximately 75% of patients in L County were presented as smear-negative. However, due to the low culture positivity rate in local laboratories, recruiting additional smear-negative cases for whole-genome sequencing proved challenging. Given that smear-negative cases have been shown to account for at least 30% of secondary transmissions (31), the inclusion of only 49 such cases in this study may lead an underestimation of the true clustering rate. Furthermore, because molecular epidemiological studies typically utilize sampling periods spanning 2 years or longer, transmission chains may be lost during surveillance efforts that are brief (as TB usually takes 6–12 months post-infection before noticeable disease manifests). Consequently, recent transmission events in L County could be far more severe than our findings. In the last, we observed the dissemination of drug-resistant strain and the accumulation of acquired drug resistance, however, only 4 clustered DR-TB strains involved the primary transmission, and serial sampling was limited to just 6 patients. Consequently, we cannot verified the confidence frequency of those events in this study. Therefore, a substantially enlarged cohort is needed to determine the true transmission pattern and drug-acquired characteristic of drug resistance TB in L County.

In conclusion, this study has demonstrated the utility of WGS for tuberculosis TB epidemiology in regions with a high burden of TB, The analysis provides insights into genomic population characteristics of TB specific to L County and indicates that a significant proportion of cases are attributable to recent transmissions— insights that are critical for developing targeted interventions against the transmission of TB. We believe that implementing continuous active case-finding strategies will significantly aid early patient identification within this area—not just focusing on key populations such as close household contacts or old population but expanding screening efforts across all residents aged over 15 years old. Furthermore, treatment regimens should strictly adhere to national guidelines alongside rigorous monitoring protocols, introducing electronic medication adherence tracking tools is also essential as effective intervention measures for enhancing chemotherapy quality and patient follow-up processes. Additionally, rapid drug susceptibility testing recommended by the WHO should be systematically implemented when diagnosing drug-resistant tuberculosis, surging that all close contacts of DR-TB patients are identified. Greater emphasis should also be placed on health monitoring among patients with drug-resistant TB to prevent further accumulation of acquired resistance and subsequent transmissions.

To enhance the characterization of transmission dynamics in future studies, the prospective investigations should focus on incorporating larger representative samples while employing robust analytical methodologies for data, including social contacts, which may ultimately contribute to the development of comprehensive models regarding transmission patterns. Enhancing the rates of favorable culture through direct-from-sputum sequencing, coupled with the implementation of ongoing surveillance, constitutes essential measures required for establishing a comprehensive network that delineates TB transmissions throughout L County. Such initiatives would yield solid scientific evidence enhancing understanding surrounding dynamic aspects related specifically toward rural mountainous regions characterized by low population mobility yet facing considerable burdens posed by tuberculosis.

## Data Availability

All Mycobacterium tuberculosis genomes data is provided by the National Microbiology Data Center. The name of the repository and accession number can be found below: https://nmdc.cn/resource/genomics/project/detail/NMDC10020193.

## Footnotes

^1^ https://www.illumina.com
